# Construction and verification of early diagnosis model of tuberculous meningitis

**DOI:** 10.3389/fcimb.2026.1829445

**Published:** 2026-06-09

**Authors:** Haoyue Hu, Minzhe Lang, Mingming Zhao, Wenwen Li, Shaoce Zhi, Peng Liu, Guangliang Hong

**Affiliations:** 1 Wenzhou Medical University, Wenzhou, China; 2 Emergency Department, Ningbo No.2 Hospital, Ningbo, China; 3 The Department of Emergency Medicine, The First Affiliated Hospital of Wenzhou Medical University, Wenzhou, Zhejiang, China; 4 The Department of Emergency Medicine, Quzhou People’s Hospital, The Quzhou Affiliated Hospital, Wenzhou Medical University, Quzhou, Zhejiang, China

**Keywords:** central nervous system infection, cerebrospinal fluid, diagnostic model, nomogram, tuberculous meningitis

## Abstract

**Objective:**

This study aims to develop a diagnostic model capable of accurately distinguishing between TBM and non-TBM in adults using basic clinical and laboratory parameters.

**Methods:**

Demographic information, clinical symptoms, imaging characteristics, and cerebrospinal fluid (CSF) parameters were collected. Consecutive patients diagnosed with TBM or non-TBM who met the inclusion and exclusion criteria were enrolled from June 2019 to June 2024 at Ningbo No.2 Hospital (training set). A random sample of 100 eligible patients from the same period at The First Affiliated Hospital of Wenzhou Medical University served as the validation set. LASSO regression with 10-fold cross-validation was applied for variable selection, generating a set of 53 variables with non-zero coefficients. These variables were then ranked by the magnitude of their coefficients, and the top 7 variables with the highest importance were retained for the final multivariate logistic regression model. Model performance, including discrimination, calibration, and clinical utility, was validated in both cohorts.

**Results:**

Multivariate logistic regression identified seizures at admission, mental and behavioral abnormalities, thalamic abnormalities on brain MRI, a positive Pandy test, cerebrospinal fluid (CSF) pressure, CSF glucose levels, and serum albumin as independent predictors of TBM (p < 0.05). The model showed promising discrimination (AUC 0.876 vs. 0.958) and good calibration in both cohorts. Decision curve analysis confirmed its clinical utility, suggesting potential to enhance diagnostic practice.

**Conclusion:**

This multivariable diagnostic model can assist clinicians in early differentiation between TBM and non-TBM using simple clinical and laboratory parameters.

## Introduction

Tuberculous meningitis (TBM) is a severe central nervous system (CNS) infection caused by *Mycobacterium tuberculosis*. It represents a fatal form of tuberculosis where the pathogen crosses the blood-brain barrier, provoking inflammation and damage to brain tissue. The infection can cause focal necrosis and vasculitis, resulting in the destruction of neural tissue and impaired brain function (Rock et al., 2008; Thwaites et al., 2013; Wilkinson et al., 2017).TBM manifests with a variety of gradually progressing clinical symptoms, such as persistent headache, fever, altered consciousness, vomiting, seizures, and cranial nerve palsies (Donovan et al., 2018). These gradual and non-specific symptoms often lead to delayed diagnoses, which increase the risk of severe complications and mortality. Diagnosis of TBM requires a comprehensive evaluation, integrating clinical presentation, cerebrospinal fluid (CSF) analysis, and imaging findings. CSF tests typically reveal favorable protein levels, decreased glucose, and lymphocytic pleocytosis (Lin et al., 2020). CT and MRI scans are valuable in identifying brain abnormalities and excluding other potential diagnoses (Kingsley et al., 1987; Dian et al., 2020). The prognosis of TBM depends on disease severity, timely diagnosis, and the overall health condition of the patient. Early diagnosis and prompt treatment are critical for minimizing neurological sequelae and reducing mortality. However, current diagnostic methods often lack sensitivity, such as smear microscopy, or are labor-intensive, like mycobacterial culture (Nhu et al., 2014; Kohli et al., 2018). New diagnostic technologies, including targeted gene sequencing, real-time polymerase chain reaction (RT-PCR), miRNA assays, and next-generation metagenomic sequencing (mNGS), offer improved accuracy but face challenges such as favorable costs, complex procedures, and limited accessibility in primary healthcare settings (Shi et al., 2023). Although Xpert MTB/RIF is currently recommended for the diagnosis of tuberculous meningitis (TBM), a negative result does not exclude TBM, and its performance remains favorably dependent on specimen quality (Cresswell et al., 2020).Therefore, there is an urgent need to develop an early diagnostic approach capable of differentiating TBM from non-TBM at the time of admission.

The nomogram, often referred to as a nomograph, has become as a predictive tool that has garnered significant attention in recent years (Iasonos et al., 2008). This tool consolidates various predictive variables into a graphical format, illustrating their influence on outcome probability within a single plane through scaled line segments. Nomograms are user-friendly, visually intuitive, and practical, which contributes to their popularity among both clinicians and patients. They have been widely utilized in oncology for prognosis prediction, notably in lung adenocarcinoma (Balachandran et al., 2015), and have recently been investigated for differential diagnosis in encephalitis (Fan, 2021). Nomograms provide individualized risk assessments based on clinical data and simple input variables (e.g., age, symptoms, and laboratory results). Since they rely on readily available clinical data without requiring advanced laboratory testing equipment, they can quickly generate relatively accurate risk estimates. This improves diagnostic efficiency and supports timely decision-making, making them particularly suitable for healthcare settings with limited resources.

## Materials and methods

**Figure 1:**
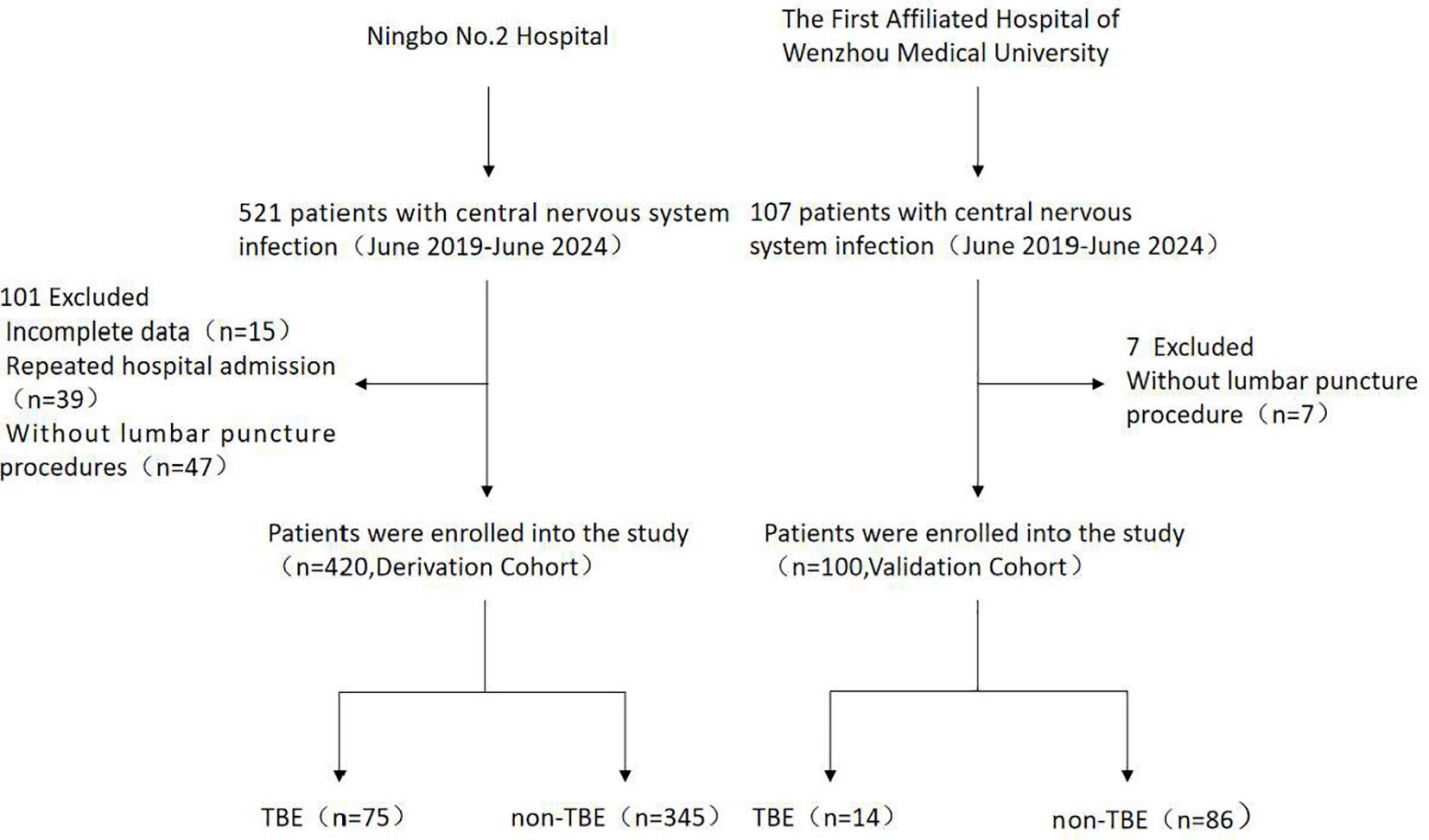
Flow diagram of the study design. A total of 420 patients with postoperative pathological diagnosis of TBM and non-TBM were included in the study of Ningbo Second Hospital, including 75 patients with TBM and 345 patients with non-TBM. The First Affiliated Hospital of Wenzhou Medical University was included in this study as a validation cohort. TBM, tuberculous meningitis.

This study included all patients diagnosed with central nervous system infections between June 2019 and June 2024 at Ningbo No.2 Hospital and the First Affiliated Hospital of Wenzhou Medical University. A total of 82 TBM cases were initially reviewed, with 7 cases excluded due to the absence of lumbar puncture procedures. Additionally, 439 non-TBM cases were analyzed, excluding 15 cases with incomplete data, 39 cases involving repeated hospital admissions, and 40 cases without lumbar puncture procedures. As a result, the training cohort consisted of 75 TBM cases and 345 non-TBM cases, divided into the TBM and non-TBM groups. Similarly, the validation cohort comprised 14 TBM cases and 86 non-TBM cases from the First Affiliated Hospital of Wenzhou Medical University. Detailed inclusion and exclusion criteria are illustrated in **Figure 1**. This study included all patients diagnosed with central nervous system infections between June 2019 and June 2024 at Ningbo No.2 Hospital and the First Affiliated Hospital of Wenzhou Medical University. A total of 82 TBM cases were initially reviewed, with 7 cases excluded due to the absence of lumbar puncture procedures. Additionally, 439 non-TBM cases were analyzed, excluding 15 cases with incomplete data, 39 cases involving repeated hospital admissions, and 40 cases without lumbar puncture procedures. As a result, the training cohort consisted of 75 TBM cases and 345 non-TBM cases, divided into the TBM and non-TBM groups. Similarly, the validation cohort comprised 14 TBM cases and 86 non-TBM cases from the First Affiliated Hospital of Wenzhou Medical University. Detailed inclusion and exclusion criteria are illustrated in **Figure 1**. All samples with missing values were excluded. Only complete cases without missing data were included for subsequent modeling, LASSO variable selection, and ROC curve analysis. The study was conducted in strict accordance with the TRIPOD (Transparent Reporting of a multivariable prediction model for Individual Prognosis or Diagnosis) statement guidelines for developing and validating multivariable prediction models (Collins et al., 2015).

Patient inclusion in the TBM group was based on the Marais unified case definition for TBM, requiring either a favorably probable or confirmed TBM diagnosis (Marais et al., 2010). A confirmed diagnosis of TBM required:

1. Symptoms or signs of brain parenchymal damage indicative of a suspected intracranial infection;
2. The presence or absence of inflammatory changes in the cerebrospinal fluid, the isolation of pathogens from the cerebrospinal fluid, blood, or abscesses, and neuroimaging examinations suggesting intracranial infection;
3. Meets one of the following conditions: a) Acid-fast bacilli staining is positive in the cerebrospinal fluid (CSF); b) Mycobacterium tuberculosis is cultured from the CSF; c) The nucleic acid amplification test for Mycobacterium tuberculosis in the CSF is positive; and the patient exhibits symptoms or signs of meningitis. Non-TBM includes viral meningitis (Solomon et al., 2012), purulent meningitis (Tauber et al., 2021), cryptococcal meningitis (Goralska et al., 2018), and autoimmune meningitis (Dubey et al., 2018).

Diagnostic criteria for non-TBM were:

1. Symptoms or signs of brain parenchymal damage indicative of intracranial infection;
2. CSF with or without inflammatory changes and the presence of viral or bacterial infection markers, or imaging evidence of intracranial infection;
3. Meeting any of the following criteria: a) Isolation or culture of pathogenic bacteria from CSF, or CSF leukocyte count > 1000×10^6^ cells/L; b) Viral isolation from CSF using NGS; c) Positive autoantibody test in CSF; d) Positive cryptococcal antigen or culture result in CSF.

Exclusion criteria included: (1) Cases of repeated hospital admissions; (2) Incomplete clinical data; (3) Patients without lumbar puncture procedures.

All procedures followed the ethical principles outlined in the Declaration of Helsinki. The study was approved by the Institutional Review Board (IRB) of both participating hospitals. Given the retrospective nature of the study, the IRB waived the requirement for written informed consent.

Continuous variables following a normal distribution are expressed as mean ± standard deviation (SD), while variables with a skewed distribution are presented as median and interquartile range (IQR). The Mann-Whitney U test was used to compare continuous variables. Categorical data are presented as frequencies and percentages, with comparisons performed using chi-square tests or Fisher’s exact tests as appropriate. Receiver Operating Characteristic (ROC) curve analysis was employed to evaluate the diagnostic ability of each laboratory test to differentiate between TBM and non-TBM.

LASSO regression was used to select variables by optimizing the regularization parameter through cross-validation, which identified the most relevant predictors for the model. These variables were then incorporated into a multivariate logistic regression model to build the diagnostic model. A nomogram was created based on the logistic regression model to visually represent the model’s predictive power, aiding clinical decision-making. Evaluate the model through the area under the curve (AUC), calibration curves, and decision curves.

All statistical analyses were conducted using SPSS software (version 27.0.0) and RStudio software (RStudio 4.3.2). All p-values were two-sided, with statistical significance set at 0.05.

## Results

### Characteristics of the training and validation cohorts

This study analyzed clinical data from 420 patients at Ningbo No.2 hospital and 100 patients at the First Affiliated Hospital of Wenzhou Medical University. Details of the enrollment process are shown in **Figure 1**. **Table 1** summarizes the demographic characteristics, clinical symptoms, and laboratory and imaging data of both the training and validation cohorts.

**Table 1:** Demographic and clinical characteristics of the study population.

Variables	Training cohort				Validation cohort			
	Total (n = 420)	non-TBM (n = 345)	TBM (n = 75)	p	Total (n = 100)	non-TBM (n = 86)	TBM (n = 14)	p
sex, male(%)	256 (61%)	216 (63%)	40 (53%)	0.173	65 (65%)	57 (66%)	8 (57%)	0.553
Meningeal irritation sign, n (%)	78 (19%)	59 (17%)	19 (25%)	0.134	18 (18%)	16 (19%)	2 (14%)	1
fever, n (%)	253 (60%)	192 (56%)	61 (81%)	< 0.001	53 (53%)	40 (47%)	13 (93%)	0.003
headache, n (%)	177 (42%)	133 (39%)	44 (59%)	0.002	27 (27%)	18 (21%)	9 (64%)	0.002
epilepsy, n (%)	54 (13%)	51 (15%)	3 (4%)	0.019	17 (17%)	16 (19%)	1 (7%)	0.453
Abnormal mental behavior, n (%)	86 (20%)	81 (23%)	5 (7%)	0.002	27 (27%)	27 (31%)	0 (0)	0.01
Memory loss, n (%)	26 (6%)	26 (8%)	0 (0%)	0.007	1 (1%)	1 (1%)	0 (0)	1
consciousness, n (%)	105 (25%)	85 (25%)	20 (27%)	0.825	24 (24%)	22 (26%)	2 (14%)	0.508
frontal lobe, n (%)	90 (21%)	62 (18%)	28 (37%)	< 0.001	15 (15%)	15 (17%)	0 (0)	0.12
temporal lobe, n (%)	69 (16%)	47 (14%)	22 (29%)	0.002	19 (19%)	17 (20%)	2 (14%)	1
parietal lobe, n (%)	49 (12%)	29 (8%)	20 (27%)	< 0.001	8 (8%)	8 (9%)	0 (0)	0.596
occipital lobe, n (%)	26 (6%)	15 (4%)	11 (15%)	0.002	7 (7%)	6 (7%)	1 (7%)	1
insula, n (%)	18 (4%)	13 (4%)	5 (7%)	0.339	14 (14%)	14 (16%)	0 (0)	0.208
basal ganglia, n (%)	27 (6%)	19 (6%)	8 (11%)	0.117	1 (1%)	0 (0)	1 (7%)	0.14
brainstem, n (%)	25 (6%)	17 (5%)	8 (11%)	0.1	2 (2%)	2 (2%)	0 (0)	1
Hippocampus, n (%)	16 (4%)	15 (4%)	1 (1%)	0.325	6 (6%)	6 (7%)	0 (0)	0.591
Cerebellum, n (%)	21 (5%)	12 (3%)	9 (12%)	0.006	1 (1%)	0 (0)	1 (7%)	0.14
thalamencephalon, n (%)	13 (3%)	9 (3%)	4 (5%)	0.261	4 (4%)	3 (3%)	1 (7%)	0.458
Centrum semiovale, n (%)	10 (2%)	8 (2%)	2 (3%)	0.695	0 (0)	0 (0)	0 (0)	
Corpus callosum, n (%)	5 (1%)	5 (1%)	0 (0%)	0.591	1 (1%)	1 (1%)	0 (0)	1
CSF Appearance, n (%)	381 (91%)	318 (92%)	63 (84%)	0.046	97 (97%)	83 (97%)	14 (100%)	1
Positive Pandy Test, n (%)	156 (37%)	107 (31%)	49 (65%)	< 0.001	53 (53%)	40 (47%)	13 (93%)	0.003
CA, n (%)	29 (7%)	27 (8%)	2 (3%)	0.178	6 (6%)	6 (7%)	0 (0)	0.591
age, Median (Q1,Q3)	49 (32, 63)	50 (34, 63)	38 (27, 56)	0.025	47 ± 17.24	46.36 ± 17.41	50.93 ± 16.17	0.345
CSF Pressure, Median (Q1,Q3)	16.5 (12.9, 21.4)	16 (12, 20)	21.4 (18.5, 21.95)	< 0.001	15 (11.38, 19.5)	14 (10.62, 17.88)	27 (21.55, 35)	< 0.001
CSF RBC, Median (Q1,Q3)	0 (0, 10)	0 (0, 10)	1 (0, 9.5)	0.186	0 (0, 8.5)	0 (0, 8)	4.5 (0, 13.75)	0.176
CSF WBC, Median (Q1,Q3)	6 (2, 75)	5 (2, 50)	45 (6, 141)	< 0.001	8.5 (2.75, 70)	8 (2, 51.5)	38.5 (6.5, 149)	0.063
CSF Glucose, Median (Q1,Q3)	3.51 (2.83, 4.06)	3.63 (3.19, 4.21)	2.35 (1.68, 2.79)	< 0.001	3.6 (3.1, 4.35)	3.7 (3.4, 4.4)	1.81 (1.4, 2.39)	< 0.001
CSF Cl, Median (Q1,Q3)	124.6 (120.18, 127.5)	125.1 (122.4, 128.1)	115 (111.45, 121.05)	< 0.001	124 (119, 127.12)	125 (121.55, 127.72)	113.25 (104.5, 116.57)	< 0.001
CSF Protein, Median (Q1,Q3)	0.62 (0.39, 1.11)	0.52 (0.35, 0.82)	1.39 (0.83, 2.15)	< 0.001	0.53 (0.43, 0.91)	0.51 (0.43, 0.82)	0.92 (0.54, 1.12)	0.084
WBC, Median (Q1,Q3)	7 (5.3, 9.4)	7.1 (5.3, 9.6)	6.7 (5.3, 8)	0.191	7.41 (6.03, 9.79)	7.69 (6.22, 10.27)	6.3 (5.96, 7.01)	0.03
Neut%, Median (Q1,Q3)	0.73 (0.64, 0.82)	0.72 (0.64, 0.81)	0.75 (0.65, 0.82)	0.349	0.72 (0.62, 0.82)	0.72 (0.62, 0.82)	0.69 (0.61, 0.8)	0.41
Lym%, Median (Q1,Q3)	0.17 (0.11, 0.25)	0.18 (0.11, 0.26)	0.15 (0.09, 0.22)	0.04	0.19 (0.1, 0.26)	0.19 (0.1, 0.25)	0.2 (0.12, 0.27)	0.502
RBC, Mean ± SD	4.3 ± 0.6	4.3 ± 0.61	4.31 ± 0.58	0.882	4.4 ± 0.58	4.4 ± 0.57	4.45 ± 0.67	0.776
HB, Median (Q1,Q3)	129 (119, 140.25)	129 (120, 142)	127 (111, 138.5)	0.056	131.8 ± 17.75	131.57 ± 17.84	133.21 ± 17.79	0.752
PLT, Median (Q1,Q3)	210.5 (162.5, 268)	209 (159, 265)	223 (168, 277.5)	0.244	206.5 (163.25, 265.5)	206.5 (164, 269.25)	200.5 (166.5, 236)	0.435
TBIL, Median (Q1,Q3)	10.4 (7.1, 13.75)	10.5 (7.1, 14)	10.3 (6.95, 13.55)	0.771	11.6 (8, 16.7)	11.85 (8.53, 16.9)	9.25 (6.25, 13)	0.245
DBIL, Median (Q1,Q3)	3.7 (2.7, 5.2)	3.7 (2.7, 5)	4.1 (2.9, 5.85)	0.062	3 (2, 4)	3 (2, 4)	2 (2, 3.75)	0.49
IBIL, Median (Q1,Q3)	6.3 (4.18, 8.7)	6.3 (4.2, 8.8)	5.9 (4.15, 7.95)	0.383	8.45 (6, 12)	9 (6, 12)	6.95 (4, 8.75)	0.053
ALB, Mean ± SD	38.9 ± 5.18	39.24 ± 5.04	37.37 ± 5.59	0.009	39.35 (36.45, 41.78)	39.15 (36.08, 41.55)	40.7 (37.33, 44.07)	0.259
AST, Median (Q1,Q3)	26 (19, 40)	26 (19, 40)	26 (20, 34)	0.959	22 (17, 30.25)	21 (17, 30)	23 (19.75, 34.5)	0.366
ALT, Median (Q1,Q3)	21 (13, 33)	21 (13, 34)	19 (13.5, 26)	0.321	19 (12.75, 30.25)	19 (13, 29.75)	18 (11, 36)	0.676
Creatinine, Median (Q1,Q3)	62.95 (51.08, 75.2)	63.3 (52.3, 75.2)	57.2 (48.75, 71.8)	0.094	68 (50.75, 79.5)	69 (54.1, 81.25)	56.5 (42, 74)	0.098
Glucose, Median (Q1,Q3)	5.96 (4.89, 6.85)	6.05 (4.88, 6.95)	5.83 (4.92, 6.5)	0.19	6.25 (5, 7.43)	5.94 (4.98, 7.85)	6.7 (6.35, 7.33)	0.132
CK, Median (Q1,Q3)	103 (56, 219.25)	108 (61, 260)	77 (42, 145.5)	< 0.001	62 (41, 172)	65 (42.25, 160.75)	49.5 (38.75, 227)	0.465
CKMB, Median (Q1,Q3)	16 (11, 23)	16 (11, 24)	15 (9, 18)	0.02	13.5 (10, 19)	14 (10, 19)	10.5 (8.14, 15)	0.133
LDH, Median (Q1,Q3)	218 (173, 258.25)	217 (173, 259)	219 (172, 250)	0.643	209.5 (174.5, 236.75)	205 (173, 231.5)	221.5 (209, 247.25)	0.141
CRP, Median (Q1,Q3)	4 (0.88, 28.58)	3.92 (0.74, 26.52)	6.39 (1.39, 32.8)	0.246	7.15 (1.3, 33.54)	5.95 (1.22, 35.04)	27 (3.6, 27)	0.468
PT, Median (Q1,Q3)	12 (11.2, 12.9)	11.9 (11.2, 12.8)	12.2 (11.35, 13)	0.254	13.5 (12.47, 14.03)	13.4 (12.43, 13.9)	14.15 (13.12, 16.75)	0.039
APTT, Median (Q1,Q3)	30.15 (27.7, 32.23)	30.2 (27.4, 32.3)	30.1 (28.5, 31.3)	0.982	33.5 (31.2, 36)	32.95 (31.02, 35.27)	36.5 (34.25, 39)	0.002
INR, Median (Q1,Q3)	1.05 (0.99, 1.13)	1.04 (0.99, 1.12)	1.07 (1, 1.15)	0.176	1.04 (0.98, 1.09)	1.03 (0.98, 1.09)	1.06 (0.98, 1.24)	0.453
D-dimer, Median (Q1,Q3)	232.5 (137,500.5)	212(130, 494)	303 (137, 545.5)	0.077	0.84 (0.35, 8.88)	0.84 (0.36, 24.5)	0.8 (0.32, 2.23)	0.374

TBM, tuberculous meningitis; CSF, cerebrospinal fluid; CA, Cancer; RBC, Red Blood Cell; WBC, White Blood Cell; Neut, Neutrophil; Lym, Lymphocyte; HB, Hemoglobin; PLT, Platelet; TBIL, Total Bilirubin; DBIL, Direct Bilirubin; IBIL, Indirect Bilirubin; ALB, Albumin; AST, Aspartate Aminotransferase; ALT, Alanine Aminotransferase; CK, Creatine Kinase; CKMB, Creatine Kinase-Myocardial Band; LDH, Lactate Dehydrogenase; CRP, C-reactive protein; PT, Prothrombin time; APTT, Activated Partial Thromboplastin Time; INR, International normalized ratio.

Univariate analysis identified several potential differentiating factors between TBM and non-TBM patients in the training cohort, including age, fever, headache, mental and behavioral abnormalities, memory impairment, abnormal signals in the frontal, temporal, parietal, occipital lobes, or cerebellum on MRI, CSF appearance, CSF pressure, CSF white cell count, positive Pandy test, and CSF chloride, protein, glucose, lymphocyte ratio, serum albumin, creatine kinase, and creatine kinase isoenzyme levels (p < 0.05).

In the validation cohort, factors such as fever, headache, mental and behavioral abnormalities, positive Pandy test, CSF pressure, CSF glucose, CSF chloride, white blood cell count, neutrophil-to-lymphocyte ratio, lymphocyte ratio, prothrombin time, and activated partial thromboplastin time were identified as potential differentiators between TBM and non-TBM (p < 0.05). Across both cohorts, the following variables were consistently significant in differentiating TBM from non-TBM: fever, headache, mental and behavioral abnormalities, positive Pandy test, CSF pressure, CSF glucose, and CSF chloride (all p < 0.05).

### Development of the diagnostic model for TBM and non-TBM

**Figure 2:**
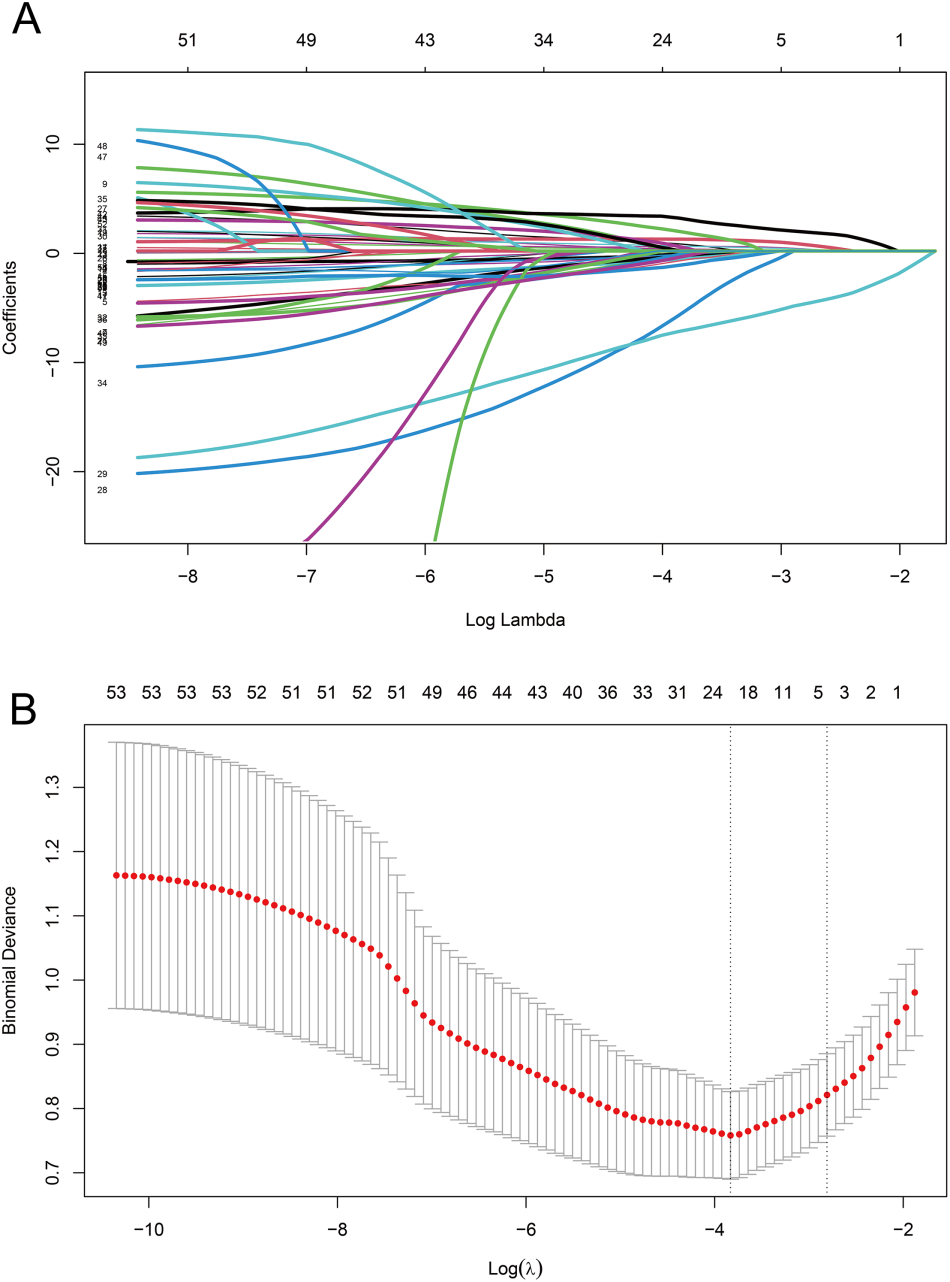
Screening of variables based on Lasso regression. **(A)** The variation characteristics of the coefficient of variables; **(B)** the selection process of the optimum value of the parameter in the Lasso regression model by cross-validation method.

Fifty-three characteristic variables included in the study were sorted by importance using lasso regression (**Figure 2**). Ultimately, the top 7 variables with the largest absolute coefficients from the LASSO regression were selected for inclusion in the model. Continuous variables included seizures at admission, mental and behavioral abnormalities, abnormal thalamic signal on brain MRI, positive Pandy test in cerebrospinal fluid, cerebrospinal fluid pressure, cerebrospinal fluid glucose, and serum albumin (see **Table 2**), which were independently associated with TBM. A logistic regression equation was derived to determine the combined probability of these 7 variables, predicting the diagnostic probability of TBM. The odds ratios (OR) from the logistic regression equation were used to determine the score for each predictor.

**Table 2:** Results of multivariable logistic regression analysis in the derivation cohort.

	B	SE	Wald	OR(95%CI)	P
(Intercept)	2.846	1.365	4.347	17.222(1.223~267.623)	0.037
Epilepsy	-1.672	0.701	5.679	0.188(0.038~0.642)	0.017
Abnormal mental behavior	-1.287	0.579	4.948	0.276(0.078~0.782)	0.026
thalamencephalon	1.852	0.797	5.408	6.375(1.239~29.436)	0.020
Pandy Test	0.863	0.318	7.352	2.37(1.271~4.447)	0.007
CSF Pressure	0.040	0.023	3.099	1.041(0.996~1.088)	0.078
CSF Glucose	-1.018	0.174	34.176	0.361(0.252~0.5)	<0.001
ALB	-0.056	0.030	3.546	0.945(0.891~1.002)	0.060

CI, confidence interval; CSF, cerebrospinal fluid; OR, odds ratio; ALB, Albumin.

### Validation of the diagnostic model

**Figure 3:**
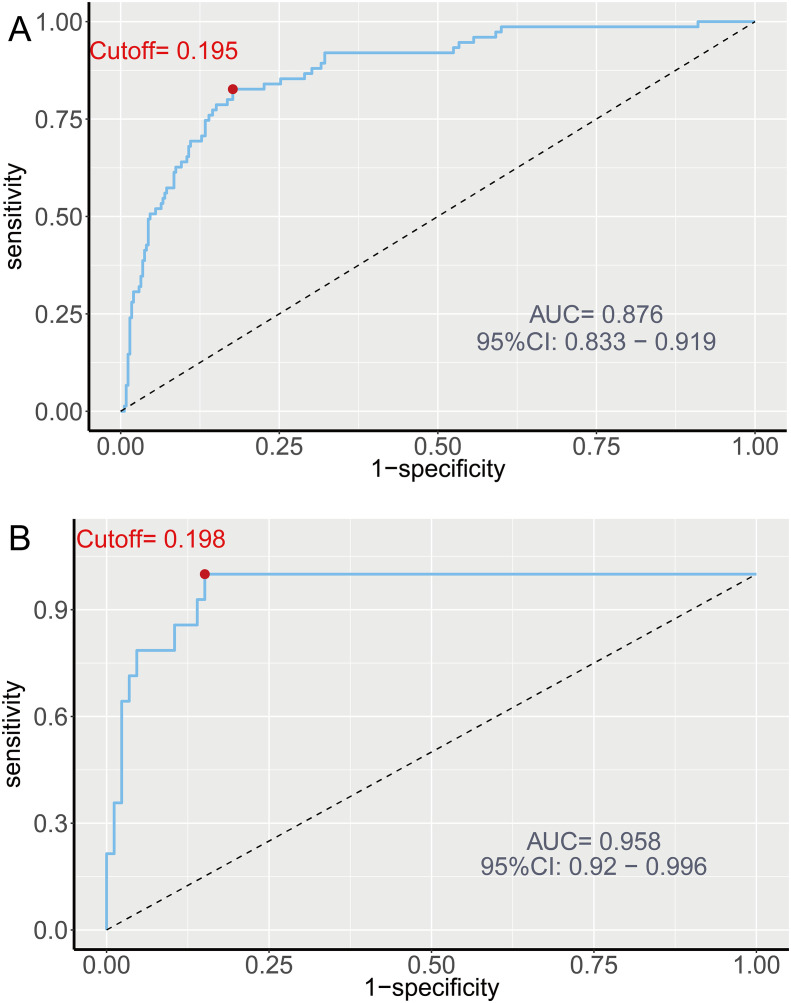
The ROC curves of the TBM diagnostic model in the derivation **(A)** and validation **(B)** cohorts. The ROC curves show the specificity and sensitivity of predicting TBM in the derivation and validation cohorts based on the model output. These values indicate the good discrimination ability of the diagnostic model. AUC, area under the curve; CI, confidence interval; ROC, receiver operating characteristic; TBM, tuberculous meningitis.

**Figure 4:**
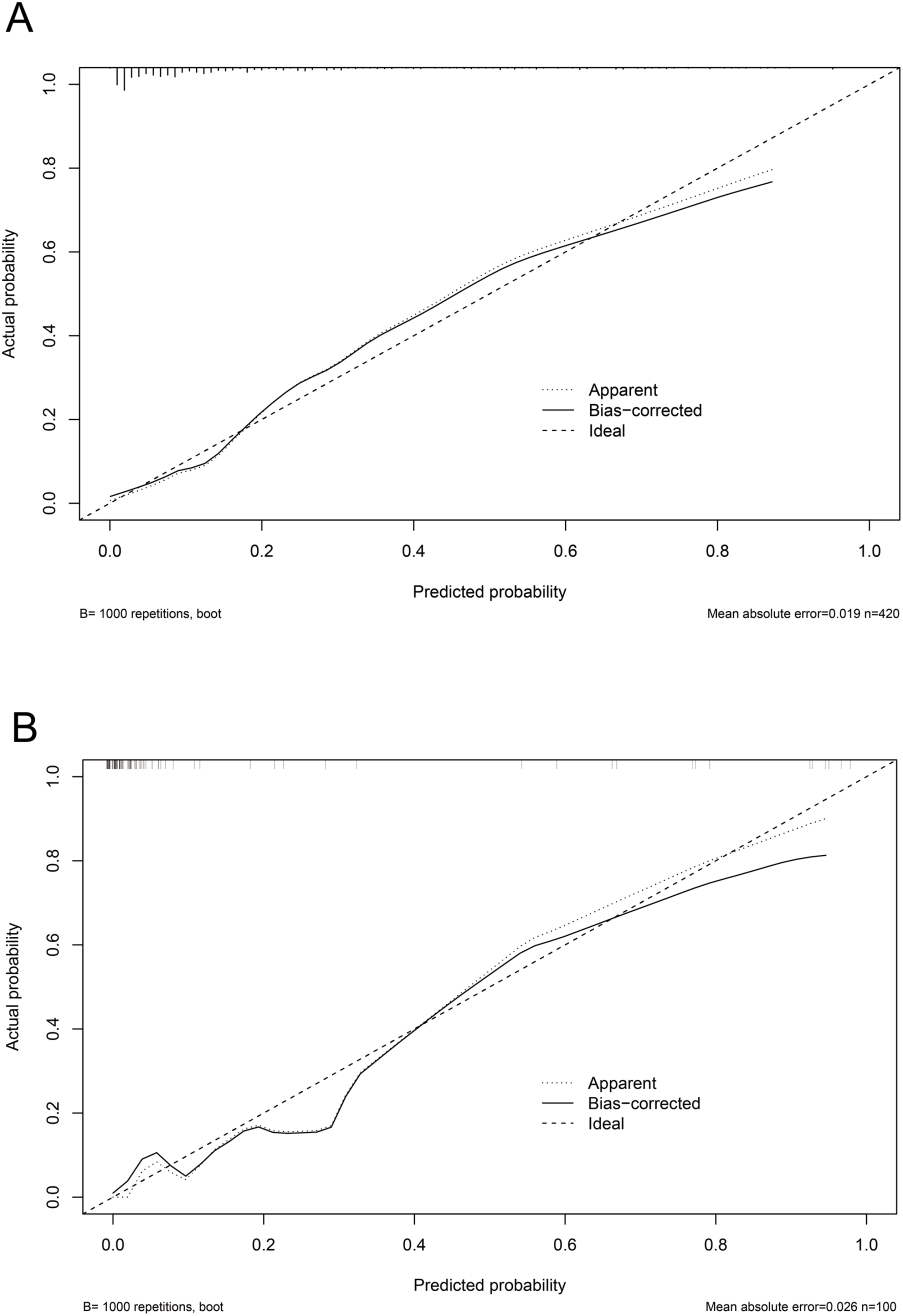
Calibration curves for the nomogram model in the derivation **(A)** and validation **(B)** cohorts. The x-axis represents the forecasted TBM risk, whereas the actual diagnosed TBM is shown on the y-axis. For each subsequent decile, the observed TBM rate in the cohort was plotted against the model prediction. The diagonal dotted line represents the ideal model with perfect prediction ability, and the solid line (bias-corrected line) represents the real performance of the nomogram. The closer the fit to the diagonal dotted line, the better the prediction ability of the nomogram. The nomogram model was promising ly calibrated in both derivation and validation cohorts.

**Figure 5:**
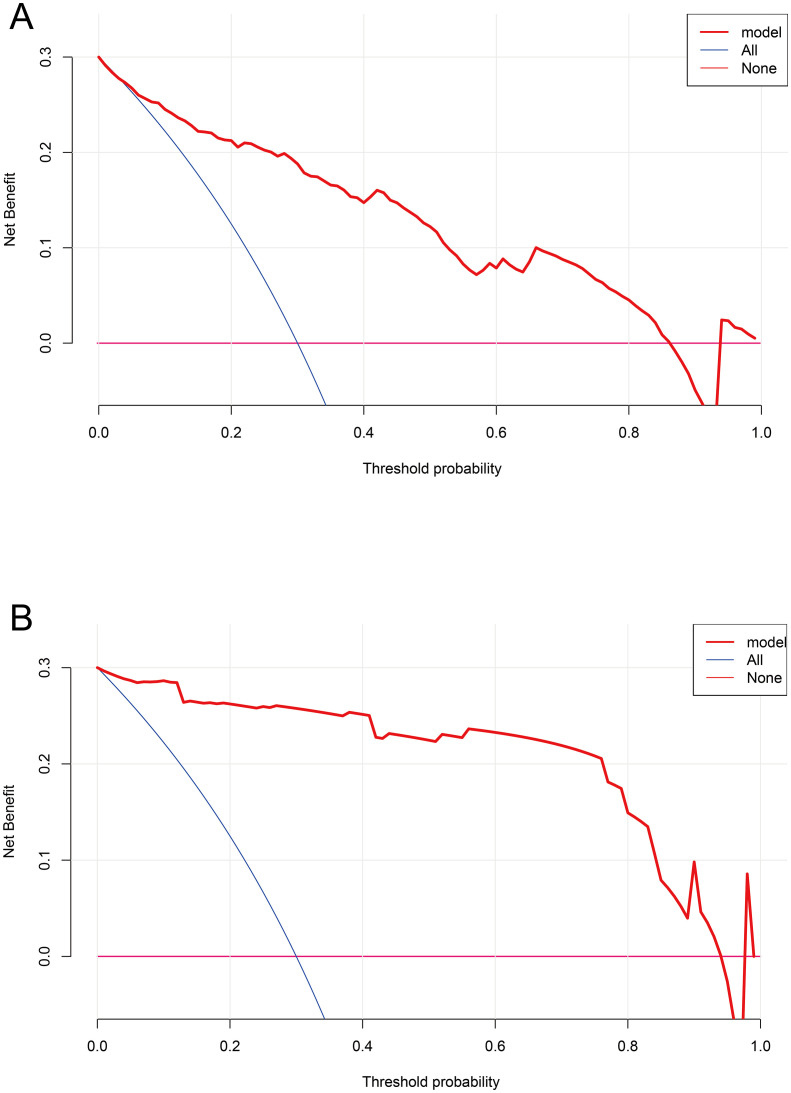
Clinical usefulness measured by decision curve analysis. The y-axis represents the net benefit. Net benefit is calculated across a range of threshold probabilities, defined as the minimum probability of disease at which further intervention would be warranted, as net benefit=sensitivity×prevalence – (1 – specificity)×(1 – prevalence)×w where w is the odds at the threshold probability. The red line represents the predicted line for a diagnostic model of tuberculous meningitis at a threshold probability ranging from 0 to 1.0. The nomogram adds net benefits compared to the treat-none (blue) and treat-all (pink) conditions in the decision curve.

**Figure 6:**
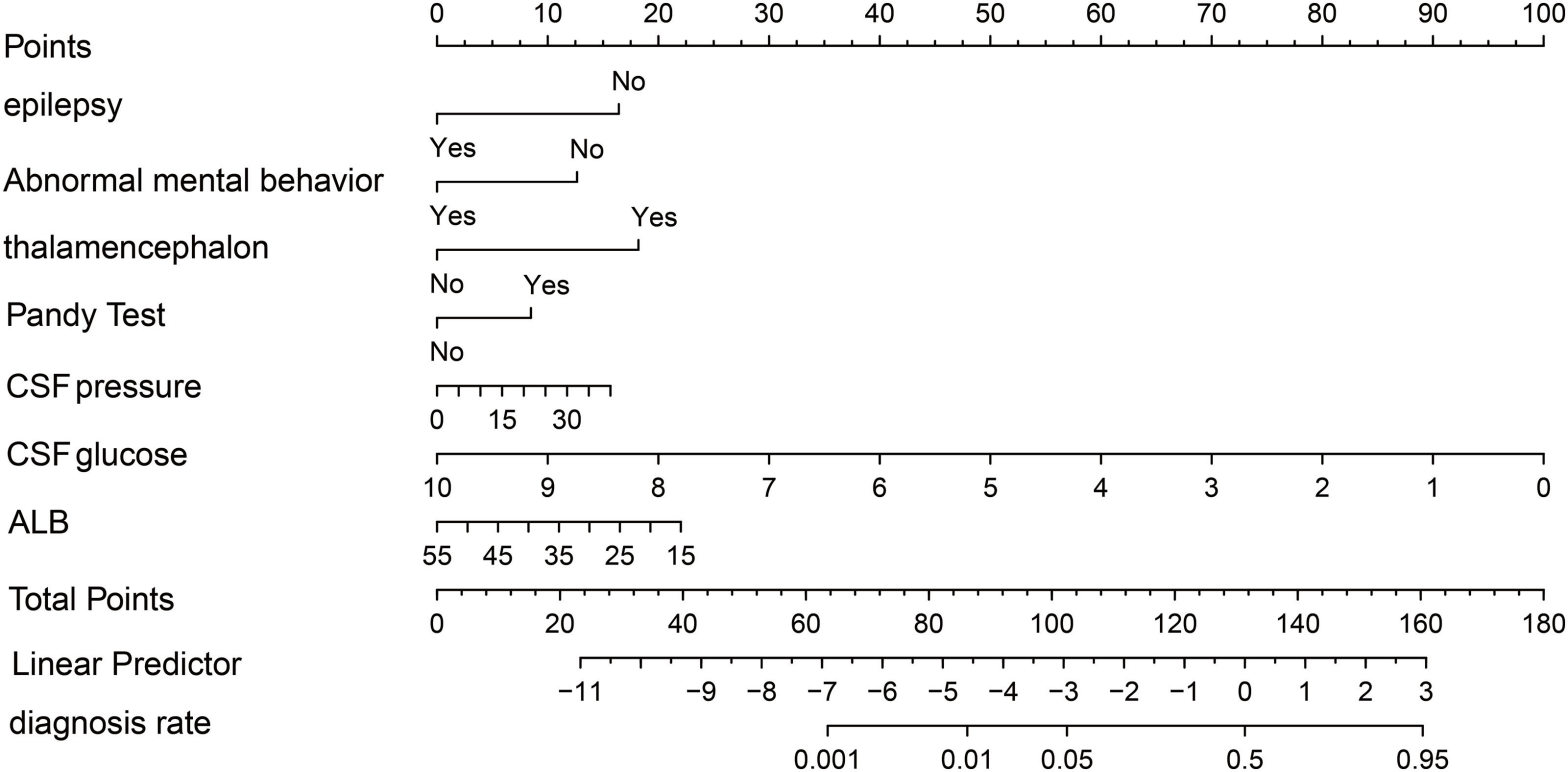
Nomogram for predicting the probability of TBM. Individual patient values were based on each variable axis of the nomogram, and the number of points obtained for each variable was determined using a line drawn downward. The sum of the points is located on the total. score axis, which corresponds on the line below to the probability of TBM. CSF, cerebrospinal fluid; TBM, Tuberculous meningitis.

**Figure 7:**
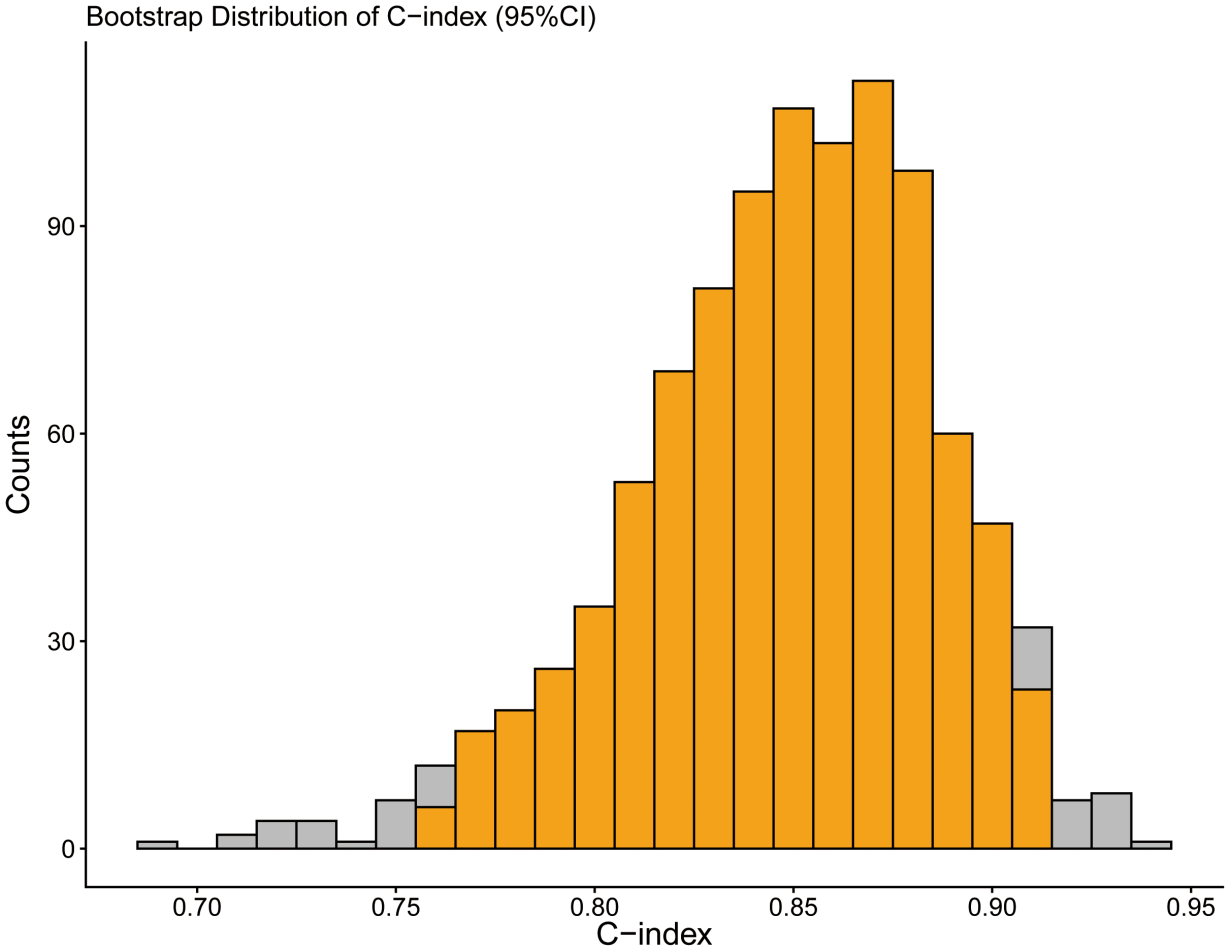
Distribution of C-index based on bootstrap resampling for internal validation. The x-axis represents the C-index value ranging from 0.70 to 0.95, and the y-axis represents the frequency count. The C-index values were concentrated at approximately 0.80–0.85 with an approximately symmetric distribution, indicating promising model stability and low risk of overfitting. C-index: concordance index, TBM, tuberculous meningitis.

**Figure 8:**
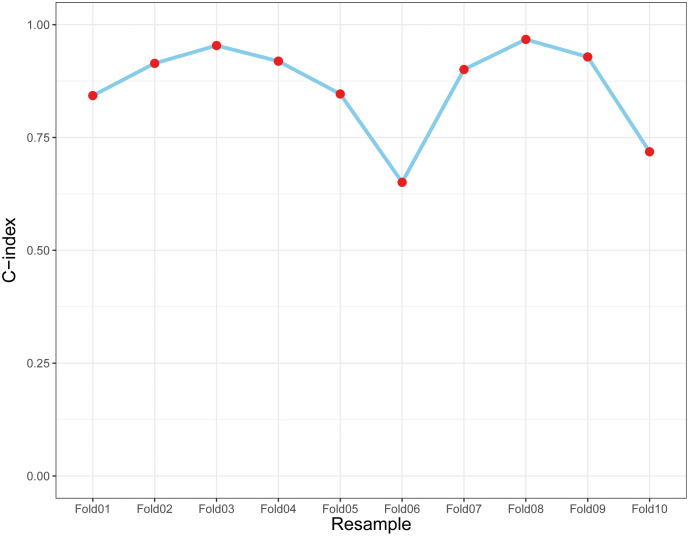
C-index values of the diagnostic model in 10-fold cross-validation. The x-axis represents each fold of 10-fold cross-validation (Fold 01 to Fold 10), and the y-axis represents the C-index value. All C-index values were above 0.75 and centered near 0.80 with minimal fluctuation, further confirming the robustness and generalizability of the TBM diagnostic model. C-index, concordance index; TBM, tuberculous meningitis.

The ROC curves and AUC values for the diagnostic model are shown in **Figure 3**. In the training cohort, the AUC was 0.876 (95% CI: 0.833-0.919), and in the validation cohort, the AUC was 0.958 (95% CI: 0.920-0.996). The optimal cutoff point for the training cohort was 0.195, achieving a sensitivity of 82.7% and a specificity of 82.3%. In the validation cohort, the optimal cutoff point was 0.198, with a sensitivity of 92.8% and a specificity of 94.1%. These results indicate that the model provides strong discrimination between TBM and non-TBM. Calibration refers to the model’s ability to accurately predict event probabilities. The calibration curve showed good agreement between the predicted probabilities and the observed outcomes across different strata. The Hosmer-Lemeshow goodness-of-fit test confirmed the model’s strong predictive performance (**Figure 4**). Decision curve analysis was performed to assess the model’s clinical utility (**Figure 5**). The decision curve demonstrated that the model provided net benefit across the entire range of threshold probabilities (0–1.0), outperforming both extreme scenarios (treating all patients versus treating none). This analysis confirmed the favorable clinical applicability of the model. To facilitate clinical use, a nomogram was developed based on seven variables (seizures on admission, mental and behavioral abnormalities, thalamic abnormalities on brain MRI, positive Pandy test, CSF pressure, CSF glucose, and serum albumin) to predict the probability of TBM (**Figure 6**). A Higher total score on the nomogram corresponds to a higher risk of TBM. To further assess model robustness and mitigate potential overoptimistic bias, we conducted internal validation via bootstrap resampling and 10-fold cross-validation. The bootstrap-based C-index ranged from 0.70 to 0.95, peaking at 0.80–0.85 (**Figure 7**). In the 10-fold cross-validation, the C-index of each fold remained above 0.75, centrally distributed around 0.80 with minor inter-fold fluctuation (**Figure 8**). Collectively, these internally corrected validation results demonstrate favorable model stability, effectively reduce overfitting concerns, and verify the satisfactory reliability and generalizability of the diagnostic model for clinical application.

## Discussion

Tuberculous meningitis (TBM) presents with atypical symptoms and low bacterial loads in cerebrospinal fluid (CSF), making early diagnosis challenging. This condition is often misdiagnosed as pyogenic, viral, or cryptococcal encephalitis. With increasing awareness of autoimmune encephalitis (AE) and improved detection of autoantibodies, the number of confirmed AE cases has risen in recent years. Studies have reported an increase in AE incidence from 0.6/100,000 to 1.2/100,000 between 2006 and 2015, indicating that AE prevalence is now comparable to that of infectious encephalitis (Dubey et al., 2018). These factors complicate the differential diagnosis of TBM.

TBM diagnosis traditionally relies on smear microscopy or culture of Mycobacterium tuberculosis, but these methods have limited sensitivity and clinical value. While the Xpert MTB/RIF assay enhances diagnostic accuracy and shortens treatment timelines, its favorable cost and equipment requirements hinder widespread use (Nhu et al., 2014; Kohli et al., 2018). Recent research has identified potential biomarkers in proteomics (Esterhuyse et al., 2015), metabolomics (Li et al., 2017), and transcriptomics (Singhania et al., 2018) for diagnosing TBM, though these markers require further validation. Consequently, clinicians often depend on medical history, clinical presentation, and basic laboratory parameters for diagnosis.

In this study, we successfully developed and validated a diagnostic model using readily available clinical indicators to distinguish between TBM and non-TBM, thereby providing a practical tool for primary care settings. In recent years, several studies have explored clinical prediction models to differentiate between TBM and non-TBM (Lee et al., 2018; Solari et al., 2018; Yang et al., 2019; Lu et al., 2021; Luo et al., 2021; International B R, 2024). These models integrate data from clinical presentation, laboratory tests, and imaging studies. Unlike previous models that focused mainly on viral and bacterial encephalitis, our model includes indicators for cryptococcal encephalitis (Beardsley et al., 2016) and autoimmune encephalitis (Dubey et al., 2018), as well as enhanced MRI features. Some prior studies utilized specialized tests (e.g., TBAg/PHA prediction) that are not feasible in economically disadvantaged or remote areas. Our model emphasizes cost-effectiveness and practical applicability in real-world clinical environments. Accurate diagnosis of potential TBM is crucial for effective treatment (Marais et al., 2010). Therefore, diagnostic models must balance cost and feasibility with diagnostic accuracy. Although our model demonstrated strong diagnostic performance, further research is needed to assess its generalizability across different populations.

In our study, we compared 53 variables (including clinical symptoms, laboratory tests, imaging findings, and CSF parameters) between adult patients with TBM and non-TBM to develop and validate a new diagnostic scoring system. Univariate analysis identified a set of potential differentiating clinical and laboratory features. Using LASSO regression and multivariate logistic regression, we defined seven independent predictors that distinguish between TBM and non-TBM: seizures on admission, mental and behavioral abnormalities, thalamic abnormalities on MRI (Kalita et al., 2019), positive Pandy test, CSF pressure, CSF glucose, and serum albumin. While Marais et al. (2010) used similar parameters to diagnose tuberculous meningitis, we adopted a different approach by developing a predictive model that combines these key variables, thereby enhancing diagnostic accuracy by considering multiple dimensions simultaneously.

Our model demonstrated that seizures and mental abnormalities were more common in non-TBM patients, consistent with previous studies. Seizures are especially prevalent in viral encephalitis, with studies showing that 30–50% of patients experience seizures, particularly those with herpes simplex virus (HSV) encephalitis (Sellner and Trinka, 2012). These seizures are likely caused by direct viral invasion, which damages brain tissue and triggers inflammation. Mental abnormalities, such as cognitive impairment, emotional instability, and psychotic symptoms (e.g., hallucinations and delusions), are also common in non-TBM patients. Jmor et al. (2008) found that cognitive dysfunction and emotional disturbances occur both in the acute and recovery phases of HSV encephalitis, aligning with our observations. These mental abnormalities likely result from inflammation affecting the frontal and temporal lobes and the limbic system, leading to disruptions in cognitive and emotional functions. Our study found that TBM tends to involve the thalamus and pituitary gland, consistent with previous research. These areas are particularly vulnerable to infection due to their central location and rich blood supply. Pituitary dysfunction, including hyponatremia, hypothyroidism, and adrenal insufficiency, is common in TBM patients (Marais et al., 2010). MRI and CT scans also frequently show inflammatory enhancement and lesions in the thalamus and basal ganglia. Calvao-Pires et al. (2022)observed low-density lesions and enhanced abnormalities in the thalamus and basal ganglia of TBM patients, supporting our findings. We also observed that serum albumin levels were often reduced in TBM patients, which is closely associated with immune suppression. TBM-induced systemic inflammation increases acute-phase protein production, leading to decreased appetite and poor nutritional intake, depleting protein stores and lowering serum albumin levels (Eckart et al., 2020). Additionally, poor nutritional status exacerbates immune deficiency. Tellez-Navarrete et al. (2021) found a significant link between malnutrition and immune dysfunction, showing that malnourished individuals are more prone to infections, which, in turn, worsens malnutrition.

In the diagnosis of TBM, CSF glucose levels and Pandy test results are critical. Previous studies have shown that low CSF glucose and a positive Pandy test are strongly associated with TBM (Lee et al., 2018; Lu et al., 2021; Liu et al., 2023). Our findings align with these results. Low glucose levels in TBM reflect the bacterium’s consumption of glucose within the CSF (Seid et al., 2023). A positive Pandy test indicates abnormal protein levels, typically caused by the inflammatory response in TBM. In our study, elevated CSF pressure also emerged as a significant predictor of TBM, likely due to inflammation and cerebral edema associated with tuberculous meningitis (Donovan et al., 2019). This finding underscores the need for further research to validate CSF pressure as a distinguishing factor for TBM.

The diagnostic model developed in this study aligns with the diagnostic rationale of The Lancet expert consensus scoring system for Tuberculous meningitis (TBM) (Marais et al., 2010), as both approaches are grounded in the following key diagnostic elements: (1) clinical manifestations (e.g., presence of impaired consciousness or meningeal irritation signs); (2) cerebrospinal fluid (CSF) parameters (including CSF albumin and opening pressure); and (3) neuroimaging features. However, our model places greater emphasis on the anatomical involvement of brain structures rather than secondary changes (e.g., hydrocephalus or tuberculomas) Highlighted in traditional scoring systems. Notably, the two diagnostic frameworks differ substantially in variable selection and clinical applicability. Our model, optimized through machine learning algorithms, requires only seven core indicators to establish a diagnosis. We therefore propose a stepwise diagnostic strategy: initial emergency screening should prioritize our rapid diagnostic model, while complex cases may benefit from supplementary evaluation using The Lancet scoring system. Compared with existing diagnostic criteria including The Lancet scoring system, our model offers distinct added value. It uses only seven routine variables, enables rapid emergency screening, and serves as an effective frontline tool to complement the more comprehensive Lancet criteria in clinical practice.

With the increasing adoption of next-generation sequencing (NGS) in infectious disease diagnostics, its utility in central nervous system tuberculosis has been preliminarily validated. One study demonstrated that CSF NGS achieved a diagnostic sensitivity of 58.8% (20/34) for TBM, which improved to 82.4% (28/34) when combined with conventional acid-fast staining and culture (Lin et al., 2021). Comparatively, our diagnostic model exhibited comparable performance (sensitivity: 82.7%; specificity: 82.3%).

In summary, our diagnostic model improves upon previous models by incorporating a broader range of clinical, laboratory, and imaging data, making it particularly suitable for resource-limited settings. In summary, our diagnostic model improves upon previous models by incorporating a broader range of clinical, laboratory, and imaging data, making it particularly suitable for resource-limited settings where rapid molecular tests such as Xpert Ultra are not readily available. By providing a practical, low-cost nomogram that can be calculated within 48 hours of admission using routine clinical data, our model enables early initiation of diagnostic anti-tuberculosis therapy without waiting for culture confirmation. The non-TBM group in our study included patients with viral, bacterial, cryptococcal, and autoimmune encephalitis, which enhances the model’s comprehensiveness. Moreover, the predictive factors in our model are based on clinically accessible data, making the approach feasible and cost-effective in diverse healthcare environments. Importantly, external validation using data from another tertiary hospital confirmed the model’s performance in terms of discrimination, calibration, and clinical applicability. The favorable AUC values for both the training and validation cohorts (**Figure 3**) and the good calibration (**Figure 4**) underscore the robustness of the model. Furthermore, decision curve analysis showed that most TBM patients benefited from the diagnostic model (**Figure 5**). The model’s visual representation as a nomogram (**Figure 6**) enhances its utility in clinical decision-making.

Several limitations should be acknowledged. First, this was a retrospective study, which may introduce potential selection bias during patient recruitment and data extraction. Second, although bootstrap resampling and 10-fold cross-validation were applied to assess model stability and minimize overfitting, the risk of overfitting cannot be fully eliminated in a predictive model based on a single regional population. Third, all patients were enrolled from medical centers in Zhejiang Province, which may restrict the generalizability of our model to other regions or countries with different epidemiological characteristics of tuberculous meningitis. Further prospective, multi-center studies with diverse geographic populations are warranted to validate and improve our model.

## Conclusion

Our study developed a novel diagnostic model based on a combination of seven variables, demonstrating promising performance in distinguishing TBM from non-TBM. This model is particularly effective in settings with limited access to pathogen detection and molecular diagnostic technologies, providing a practical tool for early diagnosis, timely intervention, and improved patient outcomes. The model’s reliance on simple clinical and laboratory parameters makes it feasible for use in primary healthcare facilities, ensuring cost-effectiveness and accessibility. However, further studies with larger and more diverse samples are needed to validate its generalizability across different regions and healthcare settings.

## Data Availability

The raw data supporting the conclusions of this article will be made available by the authors, without undue reservation.
